# Epidemic Dysentery Successfully Treated by Nux Vomica

**Published:** 1849-08

**Authors:** 


*Epidemic Dysentery successfully treated by Nux Vomica.* By W. M. Cornell, M. D.—Dr. Cornell reports having treated ten cases of this disease with Nux Vomica with uniform success. He acknowledges his indebtedness for the suggestion to Dr. Armstrong, who states that Vaux, of Ipswich, in the course of sixteen years, treated with strychnine about two hundred cases with remarkably uniform success. Dr. Cornell says, "I gave it (Nux Vomica) in the full dose of seven grains, thrice a day, to adults, and from one to three grains to children, in proportion to age. The result was most happy. Not a patient who was treated with this medicine died. It was prescribed in ten cases within three or four weeks, and all recovered. No cathartic medicine was given except teaspoonful doses of the *bitartrate of potassa*, in a few cases. * * * * * * I am far from having much confidence in *specifics* generally, but I feel constrained to say that the above named medicine altogether exceeded my expectations, and I earnestly recommend a trial of it in dysentery."—*Charleston Med. Jour.*

